# Developing a Framework for Safe AI Model Development on Sensitive Healthcare Data

**DOI:** 10.23889/ijpds.v9i5.2522

**Published:** 2024-09-18

**Authors:** Britney Le, Kristin Clemens, Salimah Shariff

**Affiliations:** 1 ICES; 2 St. Joseph's Health Care London; 3 Western University

## Objective

The Canadian Community Health Survey (CCHS) is a national cross-sectional survey, rich with household, health-related measures. Through linkage with administrative health services data, we aimed to investigate the relationship between household food-insecurity in childhood, and incident health conditions (asthma and diabetes). Linking mothers with children and children with siblings, provided a unique opportunity to expand our child cohort. 

## Approach

We linked five cycles of the CCHS with ICES administrative databases, to conduct longitudinal cohort studies of children <18 years who had a household response to the Household Food Security Survey Module (HFSSM). Three approaches were used to increase our sample of children: we included children who personally completed the HFSSM; the children of mothers who completed the HFSSM (via a unique database linking mothers with biological children); and the siblings of children who completed the HFSSM. We examined the characteristics of children from food secure and insecure households, accounting for the clustering of children living within the same home. In addition, we investigated the incidence of health outcomes using marginal time-to-event regression analysis to adjust for clinical and socio-economic confounders.

## Results and Conclusions

Using novel linkages and multiple data sources we were able to study a large cohort of children (20,023 children whose mothers completed the HFSSM, 5,199 siblings of child respondents, 8,820 children who personally responded to the survey). This creative approach built capacity to investigate household-level determinants of health in a large cohort of Canadian children.

